# Increased Retinal Metabolism Induced by Flicker in the Isolated Mouse Retina

**DOI:** 10.1523/ENEURO.0509-23.2024

**Published:** 2024-04-30

**Authors:** Robert A. Linsenmeier, Andrey V. Dmitriev

**Affiliations:** ^1^Departments of Biomedical Engineering, Northwestern University, Evanston, Illinois 60208; ^2^Neurobiology, Northwestern University, Evanston, Illinois 60208; ^3^Department of Ophthalmology, Northwestern University, Chicago, Illinois 60611

**Keywords:** isolated retina, mouse, neurovascular coupling, oxygen consumption, retinal metabolism

## Abstract

Both the retina and brain exhibit neurovascular coupling, increased blood flow during increased neural activity. In the retina increased blood flow can be evoked by flickering light, but the magnitude of the metabolic change that underlies this is not known. Local changes in oxygen consumption (QO_2_) are difficult to measure in vivo when both supply and demand are changing. Here we isolated the C57BL/6J mouse retina and supplied it with oxygen from both sides of the tissue. Microelectrode recordings of PO_2_ were made in darkness and during 20 s of high scotopic flickering light at 1 Hz. Flicker led to a PO_2_ increase in the outer retina and a decrease in the inner retina, indicating that outer retinal QO_2_ (QOR) decreased and inner retinal QO_2_ (QIR) increased. A four-layer oxygen diffusion model was fitted to PO_2_ values obtained in darkness and at the end of flicker to determine the values of QOR and QIR. QOR in flicker was 76 ± 14% (mean and SD, *n* = 10) of QOR in darkness. The increase in QIR was smaller, 6.4 ± 5.0%. These metabolic changes are likely smaller than the maximum changes, because with no regeneration of pigment in the isolated retina, we limited the illumination. Further modeling indicated that at high illumination, QIR could increase by up to 45%, which is comparable to the magnitude of flow changes. This suggests that the blood flow increase is at least roughly matched to the increased metabolic demands of activity in the retina.

## Significance Statement

Neural activity increases blood flow in the inner half of the retina as in the brain, but the underlying change in metabolism has been difficult to measure. Here we have measured the increase in metabolism (oxygen consumption, QO_2_) in the mouse retina during flicker. Flicker at high scotopic illumination increased inner retinal QO_2_ by <10% compared with darkness, considerably smaller in magnitude than the well known light–evoked decrease in QO_2_ in the outer retina under the same conditions. In the brain, the blood flow increase is larger than is required by the increase in QO_2_, but in the retina the increases in metabolism and blood flow appear to be more closely matched.

## Introduction

Neurovascular coupling, the increase in blood flow that accompanies neural activity, has been recognized for more than a century (Roy and Sherrington, 1890). In the human retina, neural activity causes an increase in the diameter of vessels in the retinal circulation (Polak et al., 2002; Garhofer et al., 2003; Riva et al., 2005) and in the vascular density of the superficial capillary plexus (Nesper et al., 2019). The resulting blood flow increase has been reviewed extensively by Riva et al. (2005). In the inner retina, responses to light increments and decrements are not balanced but are dominated by the increased firing of on-center neurons at light onset and of off-center neurons at light offset, so flickering light produces a net neural activation (Dmitriev et al., 2022). In humans and nonhuman primates, blood flow measured in the retina or optic nerve head can increase 30–58% with the optimal flickering stimulation of 8–12 Hz (Garhofer et al., 2002; Michelson et al., 2002; Riva et al., 2004, 2005; Palkovits et al., 2015). In cats the increase can be 59–250% in the optic nerve head (Riva et al., 1991; Buerk et al., 1995; Toi and Riva, 1995; Kondo et al., 1997). The vascular diameter and blood velocity increase in intact rats (Srienc et al., 2010; Kornfield and Newman, 2014), and retinal blood vessels dilate in rat isolated retina in response to 1–4 Hz flicker (Metea and Newman, 2006). Flicker-induced increases in blood flow of up to 40% have been measured in the intact mouse retina (Hanaguri et al., 2020).

When metabolic demand increases during activity, one would expect the increased need to be met either by increased blood flow or by allowing the venous oxygen saturation (S_v_O_2_) to decrease, increasing the arteriovenous oxygen difference (S_a_O_2_-S_v_O_2_), or by some combination of these strategies. However, in the brain, neural activity increases S_v_O_2_ (Ogawa et al., 1992; Hoogenraad et al., 1998; Vazquez et al., 2010; Sencan et al., 2022). Tissue PO_2_ also increases (Ances et al., 2001; Masamoto et al., 2008; Li et al., 2011; Freeman and Li, 2016; Aksenov et al., 2023). The increases in S_v_O_2_ and tissue PO_2_ indicate that the increased blood flow overcompensates for the metabolic demand for oxygen (Fox and Raichle, 1986; Ogawa et al., 1992), although perhaps not the demand for glucose (Raichle and Gusnard, 2002). The increased venous saturation during activity gives rise to the very useful BOLD signal in magnetic resonance imaging (Ogawa et al., 1992). In the human retina, the result of activity is similar in that S_v_O_2_ increases and S_a_O_2_-S_v_O_2_ decreases during flicker (Hammer et al., 2011; Felder et al., 2015; Palkovits et al., 2015). However, in rats S_a_O_2_-S_v_O_2_ increases (Shakoor et al., 2006; Blair et al., 2009; Teng et al., 2014) during flicker, as would initially have been expected. Unlike in the brain, flicker induces decreases in PO_2_ in the inner retina of rats (Lau and Linsenmeier, 2012) and in the optic nerve head of cats (Riva et al., 1991; Ahmed et al., 1994; Buerk et al., 1998). Unfortunately, in vivo PO_2_ changes cannot be used to quantify the increase in the metabolism, because PO_2_ is influenced by changes in both blood flow and metabolism.

The actual metabolic change in oxygen consumption (QO_2_) during localized activity is of interest, but it has been measured in the retina only once (Palkovits et al., 2015), by the Fick principle (i.e., multiplying blood flow by S_a_O_2_-S_v_O_2_). In order to measure the metabolic change during flicker, we took a different approach. In vivo, the local PO_2_ in the inner retina is determined by both supply, from the retinal circulation, and demand (QO_2_), as the cells use oxygen (O_2_), making it almost impossible to measure QO_2_ separately. By isolating the retina, eliminating the supply within the tissue, and providing O_2_ from perfusate at the outer (choroidal) and inner (vitreal) borders of the retina, the PO_2_ within the tissue depends only on QO_2_. We recorded PO_2_ in isolated mouse retina with O_2_-sensitive microelectrodes in darkness and light at different retinal depths and fitted a diffusion model to the data to extract QO_2_. In preparation for this work, we found previously with recordings of [K^+^]_o_ in this preparation that 1 Hz flicker produced larger changes than 10 Hz flicker (Dmitriev et al., 2022) and that four- and five-layer models of oxygen diffusion and consumption provided a good fit to profiles of PO_2_ as a function of retinal depth (Linsenmeier et al., 2023).

## Materials and Methods

**Figure 1.**
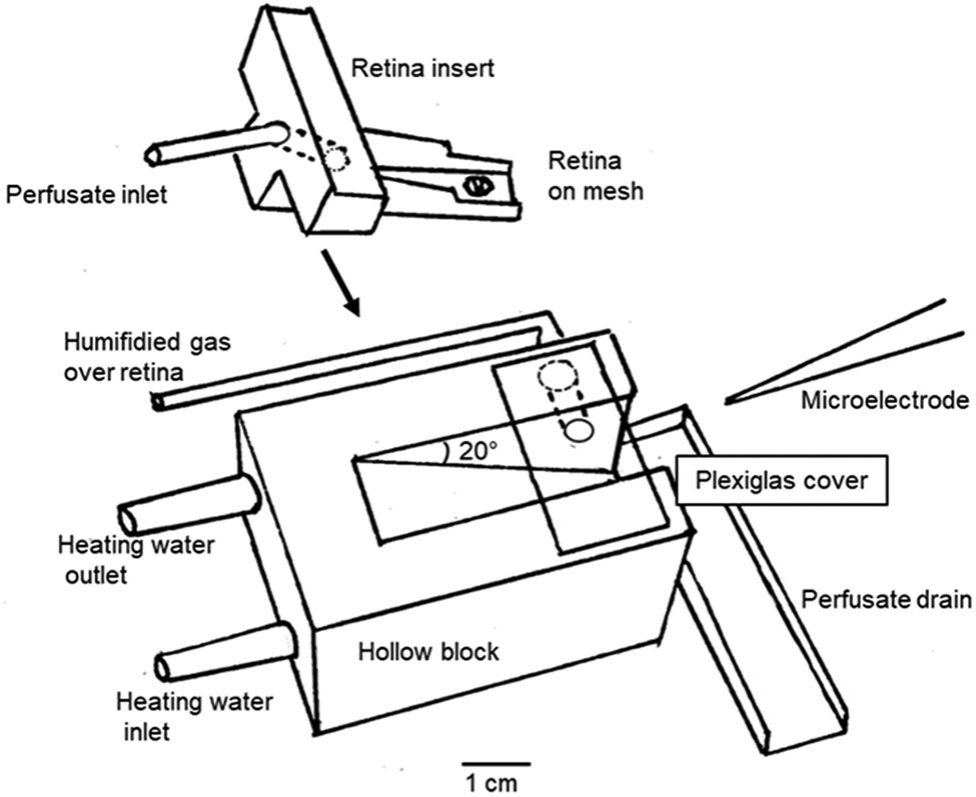
Design of the chamber for isolated mouse retina. The retina lies in the inset on nylon mesh. Perfusate flows by gravity mainly through the mesh. The inset slides into the heated block at an angle of 20° below horizontal, and correct positioning is ensured when the notches on the inset bump into the Plexiglas cover. Both the inset and the block were 3D-printed. The cover assists the distribution of gas and humidification. The microelectrode is angled at 25° below horizontal so that the retina is penetrated at a 45° angle to the retinal surface. The control of the microelectrode, with a two-axis manipulator and a Kopf hydraulic microdrive; the illumination, via a fiber-optic positioned vertically above the retina; and reference Ag/AgCl electrodes in contact with liquid in the drain are not shown. All parts are secured to a base and electrically shielded in a dark enclosure.

Animal experiments were performed in accordance with the Association for Research in Vision and Ophthalmology Statement for the Use of Animals in Ophthalmic and Vision Research and were approved by Northwestern University's Institutional Animal Care and Use Committee. The retinal preparation was as previously described (Dmitriev et al., 2021, 2022; Linsenmeier et al., 2023). Adult male C57BL/6J mice were dark-adapted for >3 h, anesthetized with 3% isoflurane in air, and killed by cervical dislocation. Retinas were isolated from the retinal pigment epithelium under dim red illumination, and the isolated retina was placed with photoreceptors up on a piece of fine CellMicroSieves nylon mesh (70 μm pore size, BioDesign) in the experimental chamber, which is shown in Figure 1. The retina was continuously perfused with a medium having the ionic and ascorbate composition of Ames’ medium (but lacking amino acids and other vitamins) at ∼36°C. The perfusate was saturated with 21% O_2_/5% CO_2_/balance N_2_ and ran mostly under the retina with only a thin film on top. Humidified gas of the same composition was passed over the top surface of the retina at 20–30 ml/min so that O_2_ was provided from both sides. This supply was enough to ensure that the entire tissue had nonzero PO_2_. Results are reported from 10 retinas from 10 animals; five were the same as in Linsenmeier et al. (2023) and five were different.

### Experimental protocol

A double-barreled O_2_–sensitive microelectrode capable of recording a current proportional to PO_2_ as well as the local electroretinogram (ERG; Linsenmeier and Yancey, 1987) was advanced through the retina from the photoreceptor side to the vitreal side. The retinas were maintained in dark adaptation except during 3 or 20 s periods of stimulation. Three second flashes of diffuse white scotopic light (0.8 lux; 3 s duration) were used to elicit ERGs, which allowed the validation of the tissue health and assisted in verifying the electrode position in the retina. At selected depths, square-wave flickering light at 1 Hz and 100% contrast (0.5 s light, 0.5 s dark) was applied for 20 s. The illumination was usually 16 lux, which is in the high scotopic range. In some cases at the same depths, steady light at half this illumination was also applied for 20 s to provide equal time-averaged illumination. At the end of experiments, after a series of short periods of illumination, low photopic (160 lux) flickering stimuli of longer duration were sometimes used.

Currents from the O_2_ barrel of the microelectrode were recorded with a Keithley Instruments 614 electrometer, low-pass filtered at 30 Hz, amplified with an instrumentation amplifier, and recorded in files on a personal computer running MATLAB (version R2013a). During calibration and experiments, the gold cathode was polarized at −0.7 V with respect to a silver/silver chloride (Ag/AgCl) reference electrode. The sensitivity of the electrodes varied from 0.06 to 0.7 pA/mmHg. The calibration was checked in the experimental chamber on a piece of nylon mesh near the retina. The voltage barrel, which contained a glass fiber, was filled with an electrolyte solution (200 mM NaCl, 10 mM HEPES, 5 mM EDTA, pH ∼7.0).

### Analysis

PO_2_s obtained at different depths in darkness just before flicker were plotted as a function of distance through the retina, with zero corresponding to the tips of the outer segments. Penetrations were at an angle to the retina, so depths were normalized to a total retinal thickness of 250 µm, the actual thickness of the mouse retina (Ferguson et al., 2013, 2014; Berger et al., 2014; Dysli et al., 2015). Values at the end of 20 s of flicker were also plotted as a function of distance. A one-dimensional, steady-state, four–layer model of oxygen diffusion and consumption (Linsenmeier et al., 2023) was fitted to the dark values. A one-dimensional model implies that there are no lateral O_2_ gradients, which is a reasonable assumption for the isolated retina of an animal with little variation of cell density with eccentricity. This model had three layers for the outer retina and one for the inner retina as in previous work (Haugh et al., 1990; Linsenmeier and Braun, 1992; Lau and Linsenmeier, 2012), described by the following equations:

$$d^{2}PO_{2}/dx^{2}=0\;\mathrm{in}\;\mathrm{Layer}\;1;\;0\;<x\;<L1,$$

$$d^{2}PO_{2}/dx^{2}=Q2/D\alpha \;\mathrm{in}\;\mathrm{Layer}\;2;\;L1\;<x\;<L2,$$

$$d^{2}PO_{2}/dx^{2}=0\;\mathrm{in}\;\mathrm{Layer}\;3;\;L2\;<x\;<L3,$$

$$d^{2}PO_{2}/dx^{2}=Q4/D\alpha \;\mathrm{in}\;\mathrm{Layer}\;4;\;L3\;<x\;<\mathrm{Lv},$$

where *x* is distance from the photoreceptor tips (*x* = 0) to the vitreous (*x* = Lv), *D* is the oxygen diffusion coefficient in the retina, *α* is the oxygen solubility, and Q2 and Q4 are the QO_2_ values in Layers 2 and 4. As previously described, *Q* = 0 in Layers 1 and 3, corresponding to the outer segments and the outer nuclear layer, because there are no mitochondria there. The observed relationship between PO_2_ and distance in those regions is a straight line, implying no consumption.

The solution to these equations, subject to matching of PO_2_ and flux at the boundaries between layers is given in Appendix 2 of Linsenmeier et al. (2023). The model parameters were iteratively adjusted to determine the set of parameters that gave the best fit to the data. The parameters that were adjusted were L1, L2, L3, Q2, and Q4, as well as the PO_2_s at the edges [PO_2_(0) = Pc and PO_2_(Lv) = Pv]. In practice, Pc and Pv were very tightly constrained by the data and were not really adjustable. Because there were fewer data points to fit than in other applications of this model (Haugh et al., 1990; Linsenmeier and Braun, 1992; Lau and Linsenmeier, 2012), we did not attempt to use the five-layer model, which included two more parameters, L4 and Q5 for the inner retina. In a paired comparison of the four- and five-layer models fitting the same data in the mouse retina, the five-layer fits had lower error but gave values for inner retinal QO_2_ (QIR) that were not different from the four-layer model (Linsenmeier et al., 2023). Also, because there were fewer data points per profile in the present work, once values had been determined for L1, L2, and L3 in darkness for a particular retina, these values were not changed in fitting the data for the same retina during flicker. Thus, only Q2 and Q4 were allowed to change between dark and light. QO_2_ for the outer retina is reported as QOR = Q2 (L2 − L1) / L3, which is a weighted average of values for the outer half of the retina and has a smaller coefficient of variation than Q2 (Linsenmeier et al., 2023). The corresponding QIR is equal to Q4.

### Statistics

Means are reported with SD. Regression analysis was used to analyze the relationship between consumption and PO_2_.

## Results

**Figure 2.**
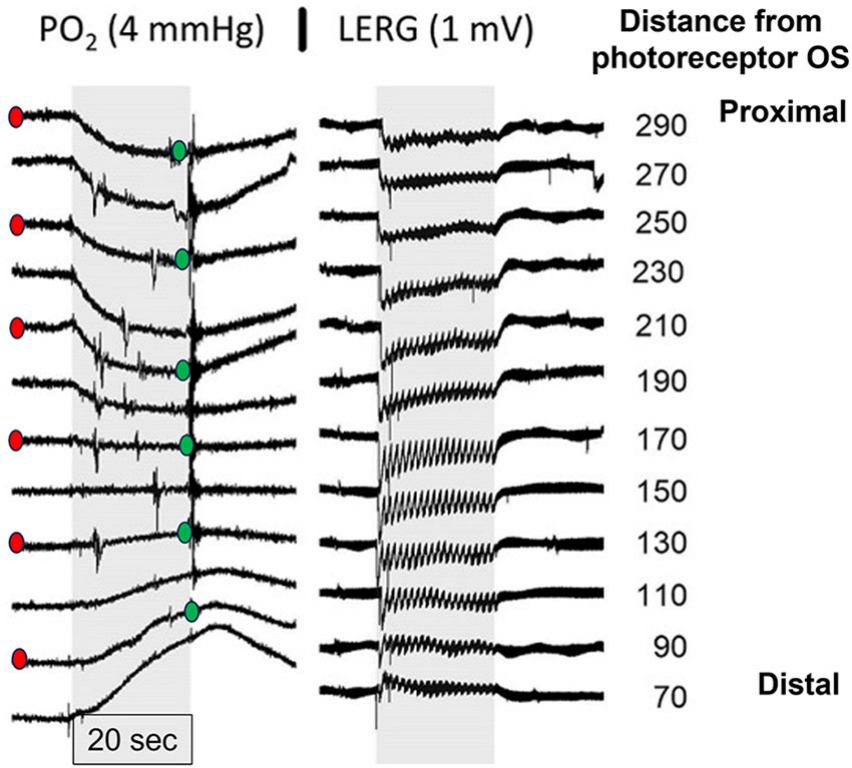
PO_2_ recordings and corresponding local ERGs (LERGs) at different depths in one isolated mouse retina. The distance is in µm from the tips of the photoreceptors. At each depth, the retina was initially in darkness, and the gray bar shows the duration of flicker. Red and green dots show PO_2_s that were used to generate profiles as in Figure 4.

Figure 2 shows recordings in one retina from distal retina at the bottom to proximal retina at the top. The left column shows changes in PO_2_ during the 20 s period of flicker. The right column shows local ERGs recorded with the voltage barrel of the microelectrode at the same depths. In distal retina, the PO_2_ increases during illumination, as is well known from in vivo recordings (Linsenmeier, 1986). The PO_2_ increase is slow in the distal retina and was not complete in 20 s, so PO_2_ continued to increase for a few seconds after the return to darkness. In the proximal retina, there is a decrease in PO_2_ that starts faster and is more complete at the end of the flicker episode. Because the PO_2_ in the perfusate at the edges of the retina does not change with light, the increase in PO_2_ in the distal retina indicates that photoreceptor metabolism decreased during flicker, and the decrease in PO_2_ in the proximal retina indicates that inner retinal metabolism increased. The inner and outer retinal events are cleanly separated, with no change at some retinal depth (here 150 µm), indicating that the inner and outer retinal events can be analyzed separately. The responses to individual flashes in the flicker stimulus are prominent in the ERG but are not visible in the PO_2_ recordings. Oxygen microelectrodes of this type respond in milliseconds (Schneiderman and Goldstick, 1978), so the absence of fluctuations in the PO_2_ recordings is caused by the dynamics of metabolism integrating the effect of flashes, rather than a slow response of the electrodes.

**Figure 3.**
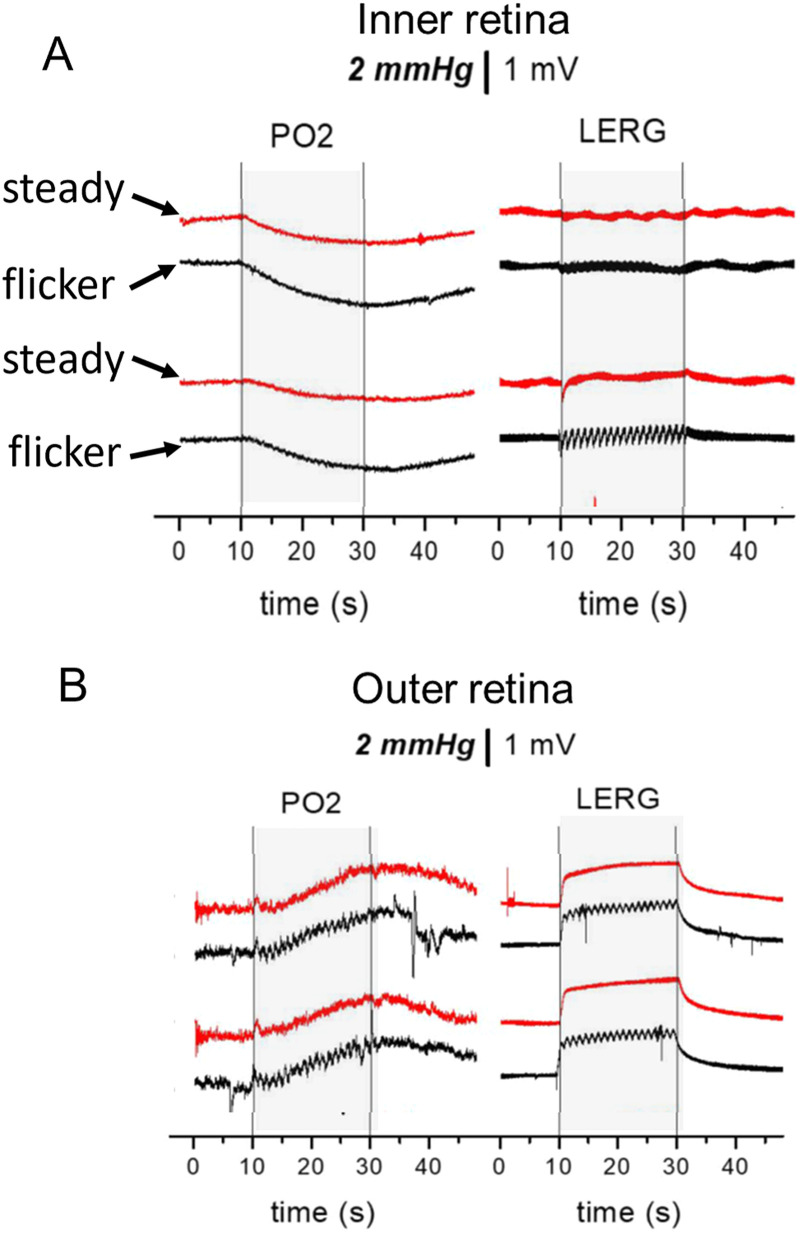
Comparison between steady light and flicker of the same time-averaged illumination at two depths in the inner retina (***A***) and two depths in the outer retina (***B***).

While our main interest was in flickering light, we also asked whether steady light would change the metabolism, and Figure 3 shows the result. For these recordings, the steady light was at half the illumination of the flicker, so that the time-averaged illumination would be equal. In distal retina, the responses to steady light and flicker were indistinguishable, indicating that, for photoreceptors, the metabolic change was the same with either stimulus. In the proximal retina, however, the PO_2_ responses to flicker were larger, but there was still a response to steady light. In one respect, this was surprising. The limited prior work has indicated that the metabolism of the inner retina is not significantly different in darkness and steady light (Bill and Sperber, 1990; Braun et al., 1995; Medrano and Fox, 1995). On the other hand, small decreases in PO_2_ with steady light in the inner retina have been seen previously in vivo (Linsenmeier, 1986; Lau and Linsenmeier, 2012). These in vivo changes could never be associated definitively with increases in the metabolism, because of the possibility that they were caused instead by decreases in blood flow, but in the isolated retina, the decrease in PO_2_ must be due to the increased metabolism. Because our focus was on changes due to flicker, the responses to steady light were not analyzed further.

**Figure 4.**
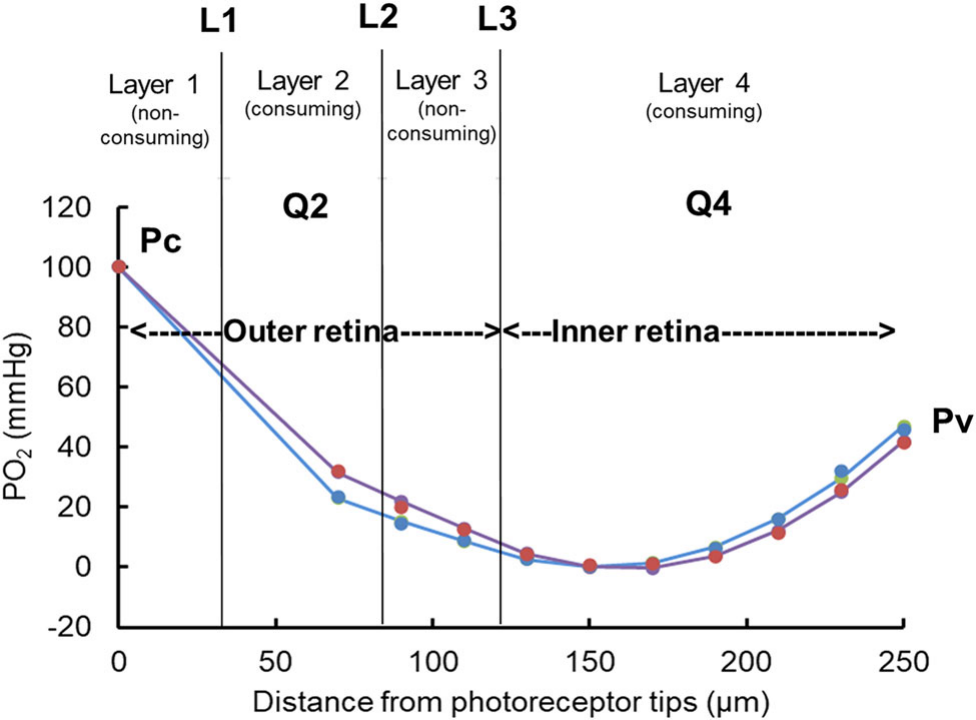
Fits of the four-layer oxygen diffusion model to values recorded in darkness and at the end of flicker. Blue symbols and fit, darkness; red symbols and fit, flicker at 1 Hz.

In order to obtain values for QO_2_, profiles of PO_2_ were constructed from datasets like that in Figure 2, using the PO_2_s just before flicker to generate a profile in darkness and the PO_2_ at the very end of flicker to generate a profile during flicker. Profiles for the data of Figure 2 are shown in Figure 4, where the symbols are from the recordings at different depths. As described in the Materials and Methods, Analysis, a four-layer model of O_2_ diffusion and consumption was fitted to the dark profiles, yielding values for Pc, Pv, L1, L2, L3, Q2, and Q4. Then the model was fitted to the data during flicker, but only Q2 and Q4 were allowed to vary. In reality the boundaries between layers would not change with light, and this was done to reduce the number of fitted parameters, given the small number of data points. Note that the fitting placed L3 at the middle of the retina, separating the retina into inner and outer halves, which corresponds to the anatomy, with photoreceptors occupying about half of the retinal thickness. The value of L3 was in the expected location across profiles: L3 = 129 ± 27 µm (*n* = 10 profiles).

**Figure 5.**
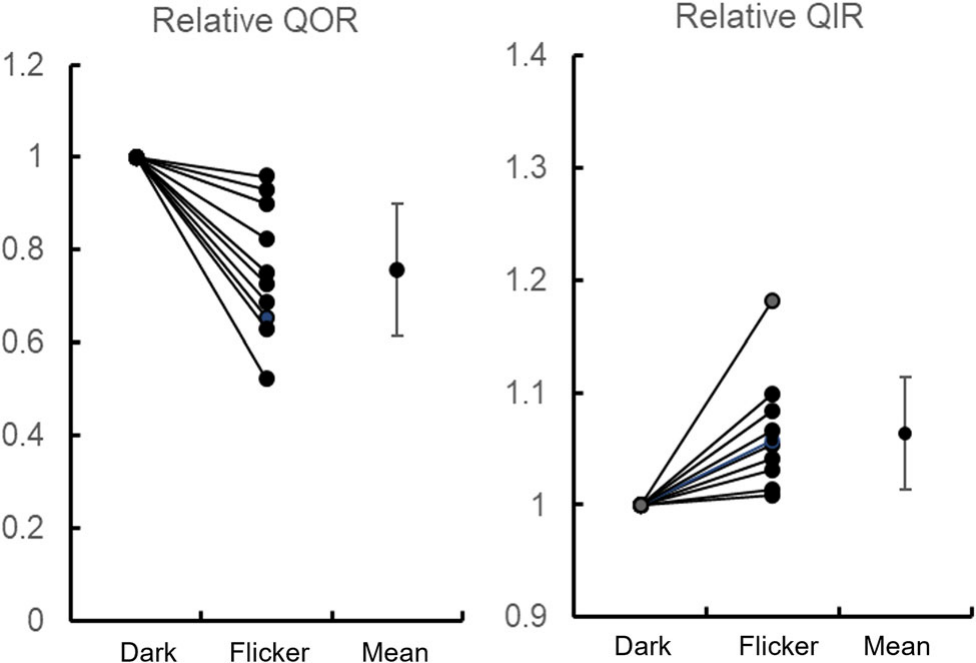
Fractional changes in QOR and QIR during flicker at 16 lux and 1 Hz for 10 retinas. QOR and QIR are normalized to 1.0 for each retina in the dark. The mean and SD for relative QOR and QIR are shown at the right of each panel. The absolute values of QOR and QIR in the dark were 1.2 ± 0.8 and 4.2 ± 2.0 ml O_2_-100 g^−1^-min^−1^.

Figure 5 shows the results of this analysis, where the dark values have been normalized to 1.0 for each retina. In every retina, there was a decrease in QOR and an increase in QIR during flicker. The decrease in QOR (0.76 ± 0.14 of the dark value during flicker) was much larger than the increase in QIR (1.06 ± 0.05 of the dark value during flicker). In only one case was the increase in QIR >10%. There was considerable variation in the magnitude of the changes in both inner and outer retina, but regression analysis showed that there was no relation between the magnitudes of the changes in each part of the retina (*p* = 0.66).

The change in QOR is smaller than might be expected from work on other animals, where there is an extensive literature on effects of light on photoreceptor metabolism (Sickel, 1972; Ames et al., 1992; Linsenmeier and Zhang, 2017). However, it should be noted that the measurements during light were made after 20 s of illumination, which does not allow the development of the full metabolic change in the outer retina, so the full change in QOR is underestimated. Figure 2 shows that the inner retinal change was complete at 20 s, so the change in QIR is a reflection of how much metabolism can change during flicker at this illumination. Neither result in Figure 5 represents the maximum possible change, because the lack of pigment regeneration in the isolated retina necessitated the use of modest illumination.

**Figure 6.**
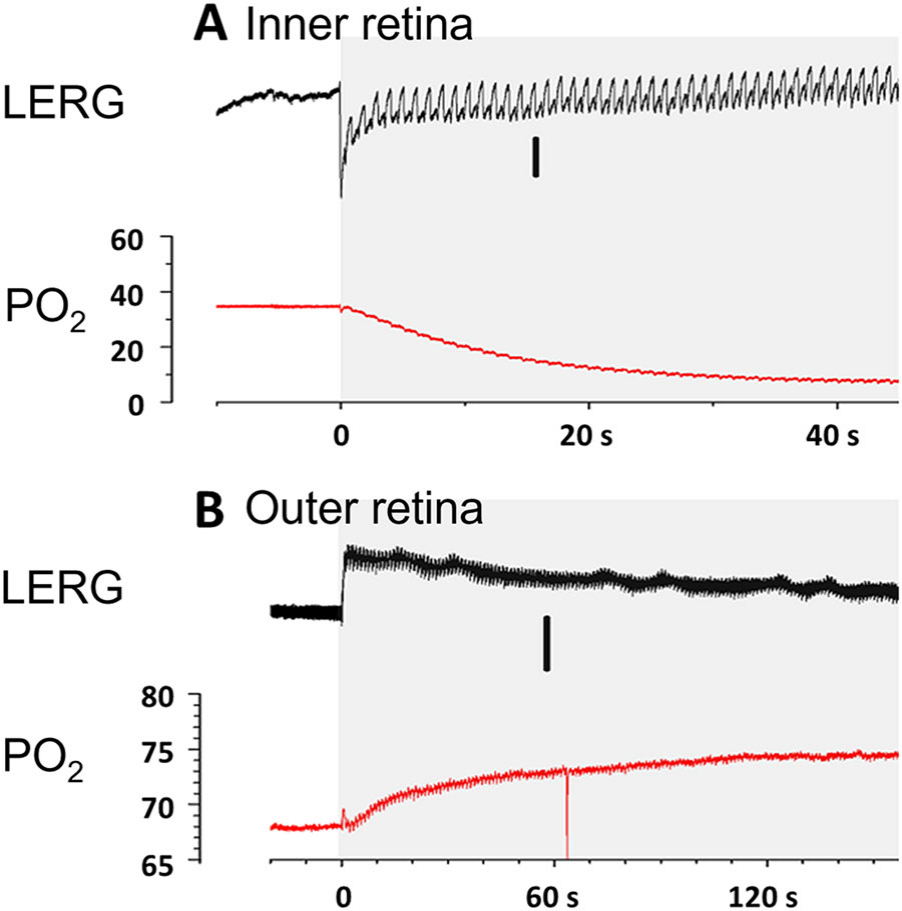
PO_2_ changes in the inner and outer retina (different retinas) for a long duration stimulus (gray bar) at 160 lux. The vertical scale bars under the LERG are 1 mV. Note the different time scales.

**Figure 7.**
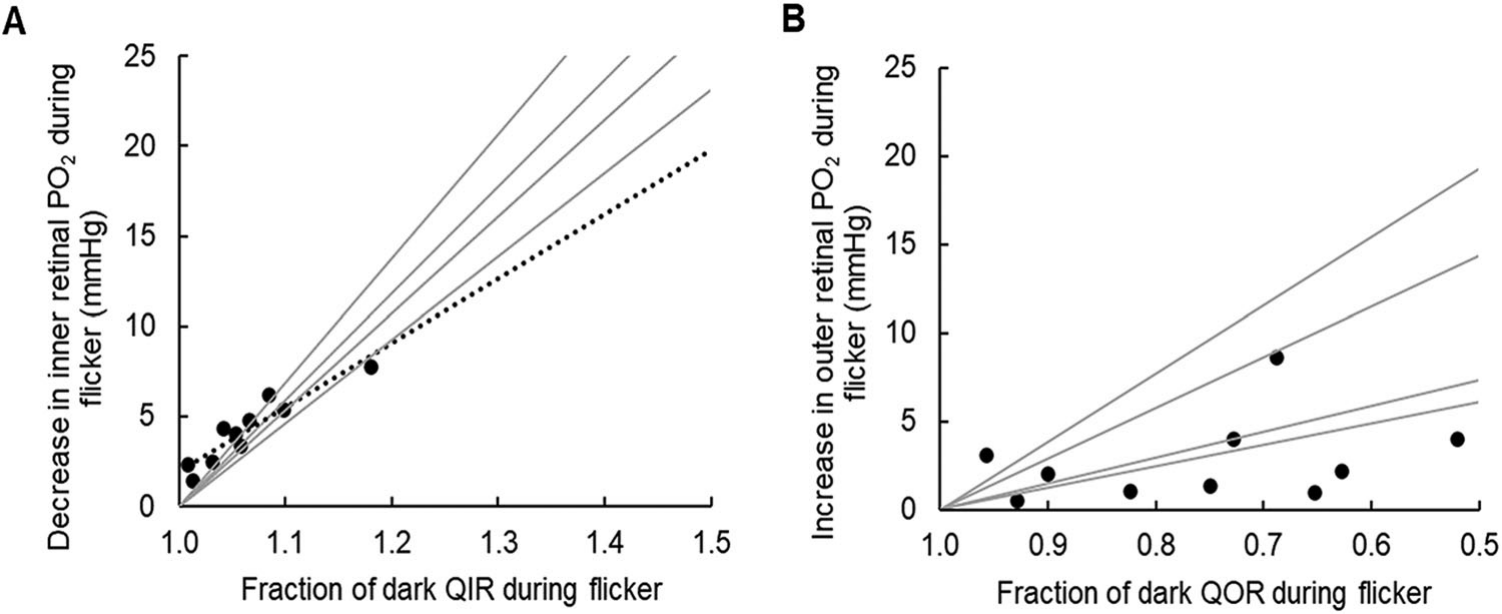
Relation between changes in consumption and PO_2_ for the inner retina (***A***) and the outer retina (***B***). Data points are for each retina. The slope of the regression in ***A*** (dotted line) was significantly different from zero (*R*^2 ^= 0.86; *p* < 0.001). For panel ***B*** the regression was not significant and is not shown. Solid lines are models for four retinas based on changing either QIR (panel ***A***) or QOR (panel ***B***) to investigate their effects on the maximum change in PO_2_ in each half of the retina.

In order to estimate the size of changes in QOR and QIR that would occur at higher illumination and longer flicker durations, one log-unit more light at a longer duration was sometimes used as the last stimulus. Figure 6 shows recordings of this type from the inner and outer retina. The PO_2_ change in the inner retina was nearly 30 mmHg. Such responses could only be obtained at one depth per retina, so the method used to derive flicker-induced changes in QIR and QOR in Figure 5 could not be used. Two approaches were used instead. First, for each retina, the largest change in inner retinal PO_2_ during flicker at the lower illumination was plotted against the change in QIR for that retina (Fig. 7*A*), and the largest outer retinal PO_2_ change was plotted against the change in QOR during flicker (Fig. 7*B*), and then a regression was used to estimate the change in QO_2_ that gave rise to larger PO_2_ changes like those in Figure 6. The relationship for the outer retinal regression was not significant (*p* = 0.36), so a PO_2_ change of a particular size, as in Figure 6*B*, could not be associated with a particular change in QOR. However, the relationship for the inner retina in Figure 7*A* was linear and significant (*R*^2 ^= 0.86; *p* < 0.001). From the regression line, a decrease of 20 mmHg in inner retinal PO_2_ during flicker would imply an increase in QOR of ∼50%. The lack of correlation for the outer retina occurs because there are more parameters (Q2, L1, L2, and L3) that go into QOR. If the outer retinal profile has a larger consuming region (L2–L1), then the predicted change in PO_2_ tends to be larger.

A second way of addressing the relation between the PO_2_ change and the Q change at higher illumination was to use the modeled parameters for each retina, either changing the value of QOR in the model with no change in QIR or changing QOR with no change in QIR and determining what this would do to the maximum PO_2_ change. This led to linear relationships for each retina, four of which are illustrated by the gray lines in Figure 7. They are a little different than the overall dashed line regression, because they were forced to go through a PO_2_ change of zero when there was no change in Q. For the inner retina, they predict that a PO_2_ change of 20 mmHg during flicker would be indicative of an increase in QIR somewhat smaller than the prediction of the overall regression line but still an increase of QIR during flicker of between 25 and 40%. This analysis also showed that for the same change in QIR and QOR, say 20%, the PO_2_ would change less in the outer retina (always <8 mmHg) than in the inner retina (always >8 mmHg). In addition, this modeling showed that the influence of changes in QOR or QIR alone propagated to a small extent through the whole retina, so the lack of change in PO_2_ at the middle of the retina (the null point) resulted from a summation of the opposing effects of flicker on the inner and outer retina, rather than an absence of an effect of flicker at the null point.

## Discussion

The PO_2_ at a point in the intact retina reflects a balance of oxygen supply and QO_2_, so it is not possible to determine how QO_2_ alone changes under different conditions. By isolating the retina and eliminating changes in supply, we were able to make the first determinations of the QO_2_ change during increased retinal activity in response to diffuse flickering light. With air-saturated perfusate, the PO_2_ at the edges of the retina was higher than it typically is in vivo, but this allowed the PO_2_ within the tissue to be maintained above zero at all points, and we could obtain normal ERGs (Dmitriev et al., 2021, 2022), providing evidence that the tissue was healthy. The decrease in QOR with light is well known (Sickel, 1972; Ames et al., 1992; Linsenmeier and Zhang, 2017) and is the same during steady and flickering light of the same time-averaged illumination. The illumination used here was lower than that in studies of the intact retina, and when this is taken into account, the percentage decrease in QOR during illumination in the mouse retina was comparable to that seen previously in other mammals (Linsenmeier, 2012; Linsenmeier et al., 2023).

In the inner retina, flickering stimuli produced a greater increase in QIR than steady light. Previous work reported no change in inner retinal metabolism with steady light (Bill and Sperber, 1990; Braun et al., 1995; Medrano and Fox, 1995; L. Wang et al., 1997), but here we could sometimes detect a small decrease in PO_2_, implying some increase in QIR, which we did not analyze further. In contrast, the decrease in PO_2_ and increase in QIR with flickering light were consistent, but averaged <10%. It was considerably smaller than the decrease in QOR (∼24%) at high scotopic illumination. Increased inner retinal metabolism during flicker was observed in a deoxyglucose study that may have used just one monkey (Bill and Sperber, 1990) and which did not attempt to quantify the change in glucose utilization, and it was also found in a human study (Palkovits et al., 2015) discussed further below. Further modeling based on our data indicated that the maximum increase in QIR during flicker would be larger with low photopic illumination, as great as an increase of 50%. The signature of this increased metabolism was also observed in recordings of inner retinal [K^+^]_o_ (Dmitriev et al., 2022) made under the same conditions. The [K^+^]_o_ increase was about twice as large under low photopic conditions as at high scotopic. Another difference between the inner and outer retina is that the QIR change from darkness to light was faster than the change in QOR. This is presumably driven by the increased pumping of the Na^+^/K^+^ ATPase at high rates of action potential firing and net depolarization of inner retinal neurons during flicker, as the [K^+^]_o_ recordings indicated, and possibly by increased neurotransmitter cycling. We did not study how the dynamics depended on illumination but note that for the outer retina, the speed of the decrease in QOR during the dark–light transition did not depend on illumination (S. Wang et al., 2010).

A question that motivated this work was how the metabolic change in the inner retina was related to the blood flow change that accompanies increased activity. We found that the change in QIR in mouse is maximally 25–50%, but it is only 6.5% at high scotopic illumination after 20 s. It is of interest that in the only study of blood flow during strong flicker in the intact mouse retina (Hanaguri et al., 2020), the blood flow increase developed slowly and was ∼10% after 60 s and 35–40% after 3 min, results that are of comparable magnitude to our QIR measurements. In the only study where both flow and QIR were measured, at an unspecified illumination, Palkovits et al. (2015) found that flow increased by 55% during a 60 s flicker episode relative to darkness and QO_2_ (which they called oxygen extraction) increased by 35%. Their results would suggest that the retina is like the brain in providing more flow than is metabolically required, but QO_2_ measured with reflectance oximetry and blood flow is not a pure measure of QIR, because some of the oxygen extracted from the retinal circulation supplies the outer retina (Linsenmeier and Zhang, 2017). If, for instance, the measured QO_2_ was 100 arbitrary units in darkness, and 80 units were devoted to inner retina, with 20 to outer retina, and QO_2_ increased to 135 units during flicker, but 120 were devoted to the inner retina, then one might conclude that QIR increased by 35%, but the increase in QIR would actually be 50% (i.e., 120/80), making the balance between the flow increase and the QIR increase almost the same, as we suggest for the mouse. A larger increase in flow than in metabolism is not consistent with measurements that indicate that tissue PO_2_ always decreases during flicker in the retina and optic nerve head (Riva et al., 1991; Ahmed et al., 1994; Buerk et al., 1998; Lau and Linsenmeier, 2012), which is another piece of evidence that during flicker, flow and QIR are more closely matched than in the brain.

In the retina, the increase in blood flow may be a little smaller than the metabolic change based on the observation in the rat that PO_2_ sometimes decreased by a few millimeters of mercury during steady or flickering illumination (Lau and Linsenmeier, 2012), whereas a matched increase in supply and demand should lead to no PO_2_ change. Blair et al. (2018), using a phosphorescence lifetime method, could not detect a difference in inner retinal PO_2_ between steady light and flicker, also suggesting that flow and metabolism were well matched when flicker causes an increase in metabolism, and with the more sensitive microelectrode methods, the difference in PO_2_ between steady light and flicker is indeed rather small (Fig. 3).

There are a few limitations of this work. First, the absolute value of QOR in the dark was lower than expected. Because mice are rod dominated with a high rod density, QOR would be expected to be similar to the value in the cat and rat, but it was about half the expected value (Linsenmeier et al., 2023). This was also observed in our earlier report of QOR in mouse (Linsenmeier et al., 2023). We attribute this to the damage to photoreceptors during isolation. Even with this lower value of QOR, the PO_2_ at the middle of the retina was close to zero, so if a preparation could be made with less photoreceptor damage, perfusate with 21% O_2_, as we used, might not be sufficient to fully supply the photoreceptors. The change with light in the outer retina was approximately as expected, but we do not know how the damage to the photoreceptors may have influenced the inner retina or the change in QIR with light. There could also have been damage during isolation to the inner retina, but the values of QIR in the dark were in the expected range. There is a high convergence from photoreceptors onto inner retinal neurons, and the ERGs were normal, so we tentatively suggest that the inner retinal values are representative of the situation in the intact retina. A second limitation is that the four-layer model was used, even though in previous work, we found that a five-layer model is preferable. This was done because the number of data points was limited here and a model with more parameters did not seem justified. In the earlier work, values for QIR were not different in a paired comparison between the four- and five-layer models. The fits of the four-layer models here had very low error per data point, so a five-layer model would be unlikely to lead to a substantial improvement or different conclusions. A final limitation is that we have only measured oxidative metabolism. It is well known that even under conditions of normal oxygenation, glycolysis contributes to the total energy production of the outer retina. Anaerobic glycolysis appears to contribute less in the inner retina (L. Wang et al., 1997), but a change in glycolytic metabolism with flicker in the inner retina cannot be ruled out and may have contributed to the increased deoxyglucose uptake observed by Bill and Sperber (1990).

In summary, the retina can be metabolically separated into two nearly equal halves, and the effects of flickering light on the metabolism of the outer and inner retina are in opposite directions, decreasing outer retinal metabolism as photoreceptors hyperpolarize with light and increasing inner retinal metabolism as second- and third-order neurons undergo net depolarization and increased firing rate. The inner and outer retina both have similar metabolic demands in darkness, but at the same illumination, flicker leads to a smaller fractional change in inner retinal metabolism than outer retinal metabolism. These differences complicate attempts to address questions in retinal metabolism that do not recognize these spatial factors. Finally, combining present and previous findings suggests that the increased retinal blood flow during retinal activation is more closely matched to the metabolic demand than is the case in the brain.

## Synthesis

Reviewing Editor: Masha Prager-Khoutorsky, McGill University Decisions are customarily a result of the Reviewing Editor and the peer reviewers coming together and discussing their recommendations until a consensus is reached. When revisions are invited, a fact-based synthesis statement explaining their decision and outlining what is needed to prepare a revision will be listed below. The following reviewer(s) agreed to reveal their identity: Wei Li. Note: If this manuscript was transferred from JNeurosci and a decision was made to accept the manuscript without peer review, a brief statement to this effect will instead be what is listed below.

Synthesis of Reviews:

Both reviewers strongly endorse the publication of this manuscript in eNeuro, recognizing its significance to the study of mammalian retinal oxygen consumption and metabolism. It would be beneficial for the author to emphasize in the discussion how, unlike in vivo conditions where oxygen is supplied by the choroid and intra-retinal vessels, in this ex vivo setup, oxygen is delivered uniformly from both sides of the retina, and whether some areas may be undersupplied. This alteration in supply will affect the diffusion dynamics within the retina for oxygen and other nutrients, a point worth stressing for a comprehensive understanding.

---

Reviewer#1

This reviewer enthusiastically supports the publication of this paper in eNeuro. The method is unique, and the results are exceptionally valuable. There are a couple of caveats that should be underscored in the discussion. It is essential that these nuances are clearly articulated to ensure the results are properly contextualized for future reference.

1. In the living animal, oxygen is provided from the choroid and several layers of intra-retinal vessels, but in this ex vivo preparation, oxygen is supplied from both external sides of the retina, which changes the diffusion dynamics in the retina for both oxygen and other nutrients.

2. The perfusate is 21% oxygen (~150 mmHg), but arterial pO2 dont exceeds 100 mmHg in mammals. Further, choroid pO2 in rodents is typically around 60 mmHg in vivo in the Linsenmeier papers (and our squirrel data). This means that the authors perfuse the retinas with solutions that are ~2.5x higher in pO2 than under in vivo physiological conditions. This explains why they get non-zero pO2s in the retinas, which the authors use as an argument for the validity of their method.

----

Reviewer#2

•Line 65 - Inserting a statement dedicated to explaining how flicker should selectively increase inner retinal neuronal activity would improve the manuscript.

•Line 69 - Rodent (mouse) retinal blood flow has indeed been measured as a function of flicker (PMID: 33188259). It seems to increase to a maximum ~40% at 12 Hz. This corresponds quite well with your data and the other data you cite, so it is worth mentioning.

•Line 104 - Dark adaptation length is not mentioned

•Line 267 - The regression analysis showing that the change in QOR does not predict the change in QIR could be worth showing, even if not significant, perhaps as a third panel of figure 5.

•Line 296 - I don't think I understand figure 7 well. In this system where retinal blood vessel flow does not determine O2 delivery, shouldn't changes in outer or inner retina pO2 always relate to QOR or QIR? This seems to be an implication of the solid grey lines in figure 7. It would seem to me that a lack of linearity corresponds to a failure of the model to properly estimate QO2. If that is true for this figure, how is it not true elsewhere? The authors are the world's leading experts in estimating QO2 from retinal pO2 profiles, so I am confident they know what they are doing. Yet like me other readers of this manuscript may not understand the inability of pO2 changes to predict QOR changes (or vice versa?) and enhancing the existing explanation would be very helpful
